# Missed Diagnosis of Syndrome of Inappropriate Antidiuretic Hormone Secretion (SIADH) as a Paraneoplastic Syndrome in Small Cell Lung Cancer: A Case Report

**DOI:** 10.7759/cureus.86678

**Published:** 2025-06-24

**Authors:** Mohamed Naas, Benjamin K Linkous, Filip Ptak, Angeli J Canekeratne, Samerah Razuman

**Affiliations:** 1 Radiology, Florida State University College of Medicine, Tallahassee, USA; 2 Internal Medicine, Florida State University College of Medicine, Tallahassee, USA; 3 Nephrology, Florida State University College of Medicine, Tallahassee, USA

**Keywords:** alcohol use, delayed diagnosis, imaging findings, paraneoplastic syndromes, repeat scanning, small-cell lung cancer, syndrome of inappropriate secretion of antidiuretic hormone (siadh)

## Abstract

Small cell lung cancer (SCLC) is an aggressive malignancy frequently associated with paraneoplastic syndromes, including the syndrome of inappropriate antidiuretic hormone secretion (SIADH). These syndromes usually happen prior to tumor detection and can lead to delayed diagnosis, especially when initial imaging is inconclusive. Here we report the case of a 56-year-old female with a history of chronic tobacco and alcohol use who presented to the emergency department multiple times over two months with symptoms of fatigue, dizziness, and hyponatremia. After several hospital encounters and repeated chest X-rays, an eventual chest CT scan revealed a right hilar mass. The patient was subsequently diagnosed via endobronchial biopsy and started on chemotherapy. This case highlights the diagnostic challenge of paraneoplastic SIADH in SCLC with imaging, especially in patients with confounding risk factors for hyponatremia. Initial negative imaging delayed diagnosis, underscoring the limitations of chest X-rays and the need for high clinical suspicion in high-risk populations. Early CT imaging may expedite diagnosis, reduce delays in treatment, and improve clinical outcomes.

## Introduction

Lung cancer remains the leading cause of cancer-related death in the United States, accounting for approximately 125,000 deaths annually and nearly 20% of all cancer deaths [1,2]. Small cell lung cancer (SCLC), an aggressive subtype, comprises about 15% of all lung cancer cases [1-3]. While smoking is the main risk factor for lung cancer, many cases go undetected until the cancer is advanced due to the often nonspecific nature of early symptoms [2,4]. SCLC is known for its association with paraneoplastic syndromes, such as syndrome of inappropriate antidiuretic hormone secretion (SIADH), Lambert-Eaton myasthenic syndrome, and Cushing syndrome, which can further convolute the diagnosis and management process [1].

SIADH is a well-known cause of hyponatremia, often associated with malignancies, medications, and central nervous system disorders [5]. Among malignancies, SCLC is the most recognizable cause of SIADH due to secretion of antidiuretic hormone (ADH) [6,7]. Hyponatremia in SIADH typically develops insidiously and manifests with nonspecific symptoms such as fatigue, dizziness, and generalized weakness, which can mask the underlying cause and contribute to diagnostic delays [5]. Given that early-stage SCLC is often asymptomatic or presents with vague constitutional complaints, SIADH may serve as an early clinical and diagnostic clue in affected individuals [8]. However, failure to promptly recognize hyponatremia secondary to SIADH as a potential paraneoplastic syndrome can result in missed opportunities for early cancer diagnosis. These syndromes can present prior to detection of the malignancy, leading to multiple healthcare encounters before a definite diagnosis is reached, as seen with our case. The diagnostic complexity increases in patients with co-morbid conditions or other risk factors for hyponatremia, such as chronic alcohol use and medication effects, which can initially shift attention away from a malignancy [4]. This challenge underscores the importance of maintaining a high index of suspicion, particularly in individuals with persistent or recurrent SIADH who belong to high-risk groups, such as long-term smokers.

This case report highlights a 56-year-old woman with a history of heavy tobacco and alcohol use who presented to the emergency department (ED) four times over two months with nonspecific symptoms such as fatigue and cough. Despite multiple normal chest X-rays, a subsequent contrast-enhanced CT scan revealed a right hilar mass, leading to the diagnosis of SCLC. This case emphasizes the need for early consideration of paraneoplastic syndromes in cases of unexplained hyponatremia, particularly in high-risk individuals, and the critical role of timely imaging in facilitating prompt diagnosis and intervention.

## Case presentation

A 56-year-old female with a history of hypertension, dyslipidemia, major depressive disorder, and chronic alcohol use presented to the ED on multiple occasions over a two-month period with symptoms primarily related to hyponatremia and fatigue. The patient's social history was notable for significant tobacco use with a 40-pack-year history and chronic alcohol consumption of approximately five beers daily.

Initial presentation (initial visit)

**Figure 1:**
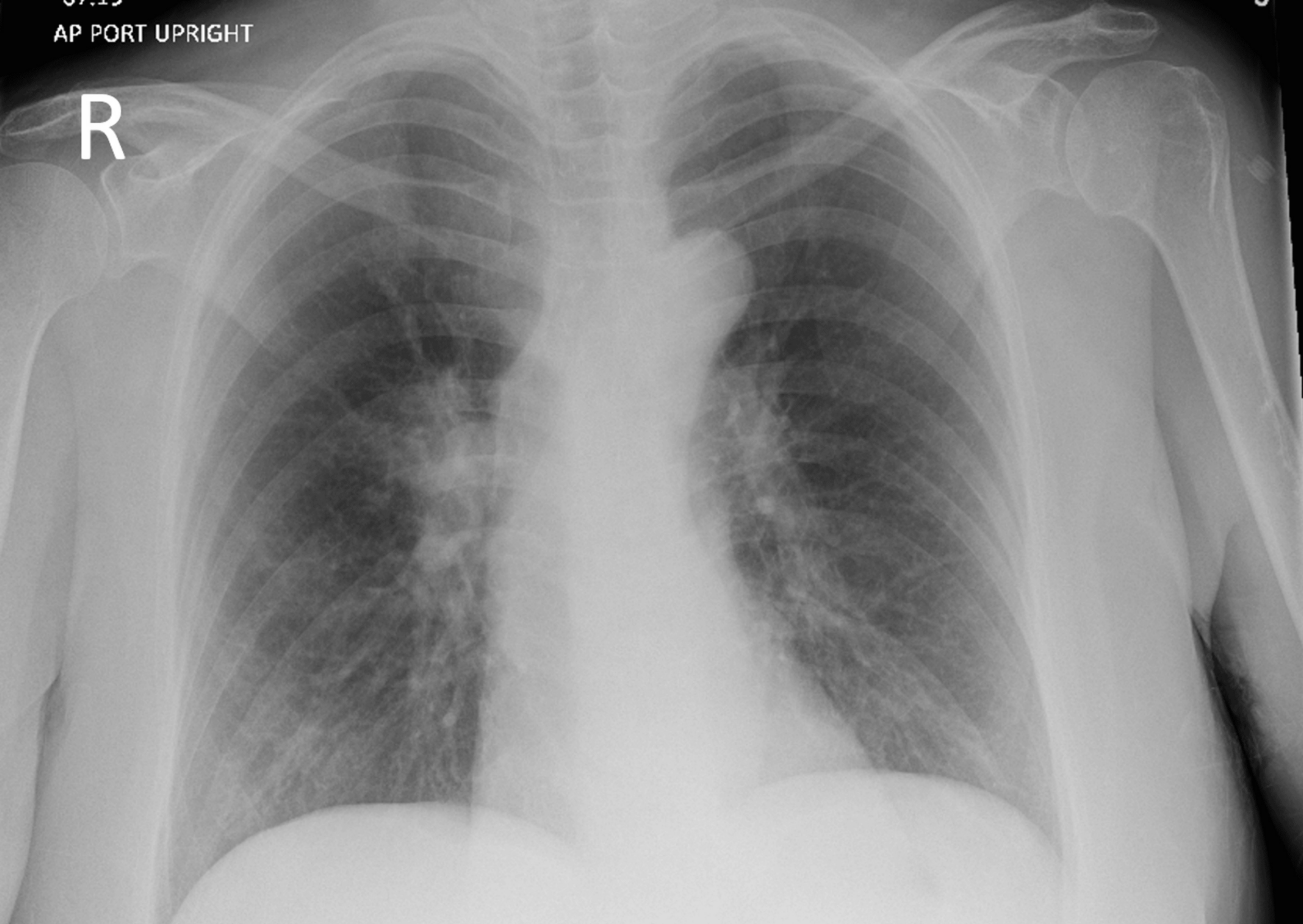
Chest X-ray from the first ED admission. Initial chest X-ray showing peribronchial cuffing and bilateral hilar prominence, findings attributed to acute bronchitis or underlying airway disease.

The patient first presented to the ED with two weeks of dyspnea and roughly four to six hours of sensation of her throat closing. She denied chest pain, wheezing, abdominal pain, nausea, vomiting, rash, leg swelling, or cough. On physical examination, she was alert and in no acute distress. Her vital signs were notable for a blood pressure of 172/71 mmHg, heart rate of 69 bpm, and oxygen saturation of 100% on room air. Laboratory results revealed a critically low sodium level of 114 mmol/L (reference range: 135-146 mmol/L), without any documented or patient-reported history of hyponatremia (Table 1). An initial chest X-ray showed peribronchial cuffing and bilateral hilar prominence, which was attributed to acute bronchitis or airway disease (Figure 1). No serum osmolality, urine osmolality, or urine sodium studies were obtained during this initial visit. The etiology of the hyponatremia was not further investigated at this time, and it was presumed to be secondary to alcohol-related causes. She was treated in the ED with IV normal saline. The patient felt better with IV fluids and decided to leave the ED against medical advice.

**Table 1:** Summary of key laboratory findings across emergency department visits. WBC: white blood cell; RBC: red blood cell; HGB: hemoglobin; HCT: hematocrit; MCV: mean corpuscular volume; MCH: mean corpuscular hemoglobin; MCHC: mean corpuscular hemoglobin concentration; RDW-CV: red cell distribution width-coefficient of variation; MPV: mean platelet volume; PLT: platelets; BUN: blood urea nitrogen; NT Pro BNP: N-terminal pro B-type natriuretic peptide; GFR Non-AA: glomerular filtration rate, non-African American; AST: aspartate aminotransferase; ALT: alanine aminotransferase

Lab Test	Reference Range	ER Visit 1	ER Visit 2	ER Visit 3	ER Visit 4	Inpatient
WBC	3.8-11.1 x 10^3/uL	-	3.59	6.68	6.38	5.32
RBC	3.64-5.41 x 10^6/uL	-	3.67	3.26	3.37	3.19
HGB	12.0-16.0 g/dL	-	10.5	10.6	10.6	9.9
HCT	34.3-46.7%	-	36.4	30.4	31.6	30.2
MCV	80.1-99.4 fL	-	99.2	93.3	93.8	94.7
MCH	26.4-33.5 pg	-	32.2	32.2	31.5	31.0
MCHC	31.1-35.5 g/dL	-	32.4	34.5	33.5	32.8
RDW-CV	11.5-14.5%	-	12.3	13.0	12.8	12.7
MPV	9.4-12.3 fL	-	9.0	9.4	8.7	8.7
PLT	139-391 x 10^3/uL	-	172	272	380	322
Glucose	70-99 mg/dL	-	121	102	104	96
BUN	7-18 mg/dL	-	9	12	9	8
Creatinine	0.6-1.3 mg/dL	-	0.78	0.51	0.59	0.51
BUN/Creatinine	5-20	-	11.5	23.5	15.2	15.6
Sodium	135-146 mmol/L	114	128	128	136	131
Potassium	3.5-5.0 mmol/L	-	4.0	4.3	3.7	3.3
Chloride	98-109 mmol/L	-	98	97	99	98
CO2	17.0-34.0 mmol/L	-	18	20	25	24
Anion Gap	8-12 mEq/L	-	12	11	12	9
Calcium	8.5-10.1 mg/dL	-	8.4	8.9	9.9	8.5
Bilirubin, Total	0.1-1.2 mg/dL	-	1.0	1.8	0.4	-
Protein, Total	6.0-8.3 g/dL	-	6.2	5.9	6.6	-
Albumin	3.5-5.5 g/dL	-	3.7	3.0	3.7	-
Creatine Kinase	25-200 IU/L	-	61	-	26	-
NT Pro BNP	<125 pg/dL	-	2200	110	1384	-
GFR Non-AA	>60	-	89	-	106	110
AST	10-40 U/L	-	427	50	21	-
ALT	7-56 U/L	-	302	48	12	-
Alkaline Phosphate	44-147 U/L	-	535	891	175	-
Lactate	0.7-2.0 mmol/L	-	1.8	0.9	-	-

Subsequent visit #1 (ER visit #2)

**Figure 2:**
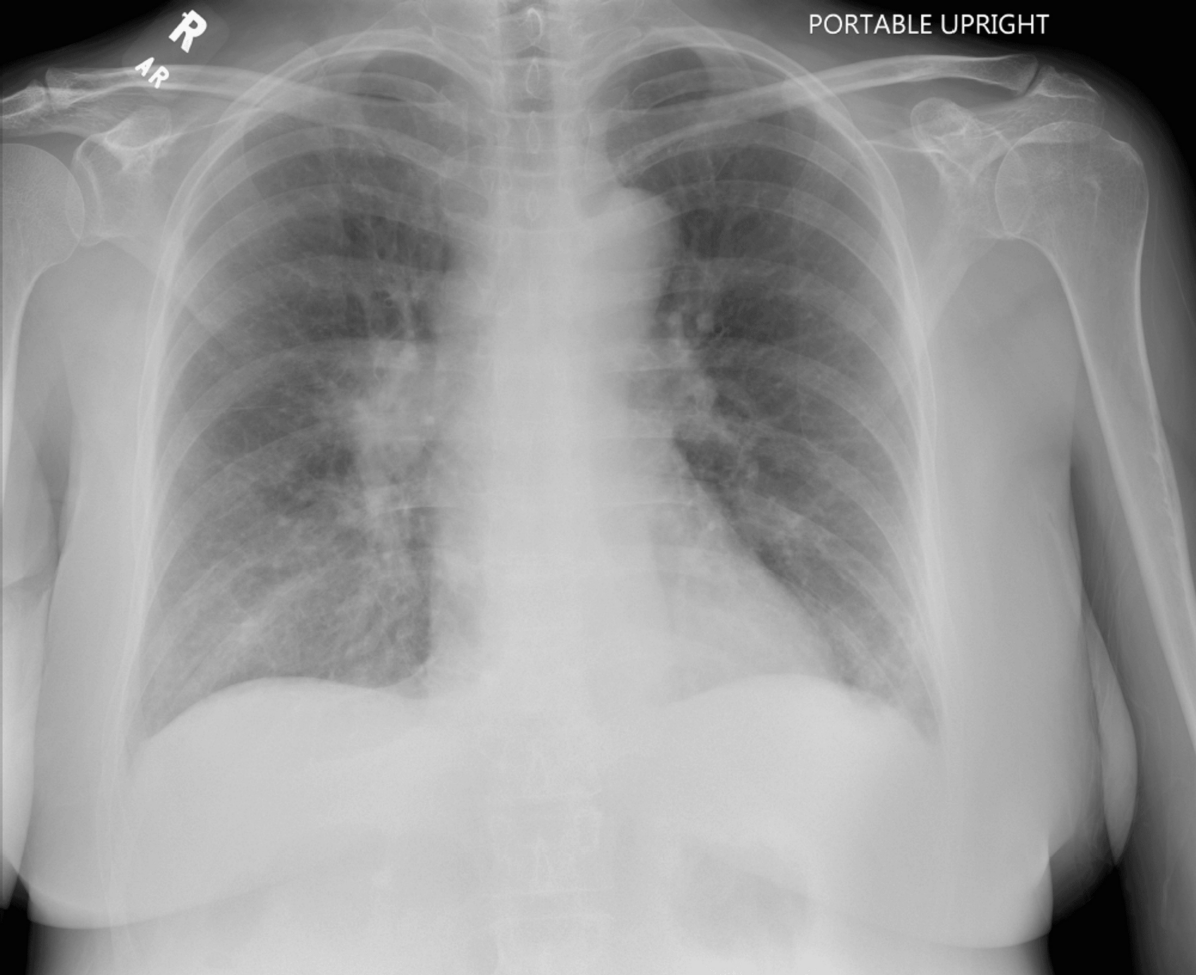
Chest X-ray from the second ED admission. Repeat chest X-ray demonstrating no evidence of acute cardiopulmonary disease, supporting no clinical change from prior airway findings.

Two weeks later, the patient returned to a separate ED complaining of dizziness, hypotension, and persistent malaise. She reported her prior hospitalization at another hospital for hyponatremia. On physical examination, she appeared fatigued but was alert and oriented. Her blood pressure was 119/78 mmHg, heart rate 92 bpm, respiratory rate 26 breaths per minute, and oxygen saturation 98%. Laboratory findings included an improved sodium level of 128 mmol/L (reference range: 135-146 mmol/L). However, no additional tests such as serum osmolality, urine osmolality, or urine sodium were obtained during this visit to further evaluate the cause of the hyponatremia. Her liver enzyme abnormalities were presumed to be secondary to chronic alcohol use. No liver ultrasound or additional hepatic imaging was performed at this time. She also had mild anemia with a hemoglobin level of 10.5 g/dL (reference range: 12.0-16.0 g/dL) (Table 1). A repeat chest X-ray showed no acute cardiopulmonary disease (Figure 2). Given her persistently low sodium and recurrent symptoms, the patient was advised to initiate outpatient fluid restriction and oral sodium supplementation. Although formal diagnostic testing for SIADH had not been completed, the clinical picture raised concern for possible euvolemic hyponatremia, prompting referral to nephrology.

Subsequent visit #2 (ER visit #3)

**Figure 3:**
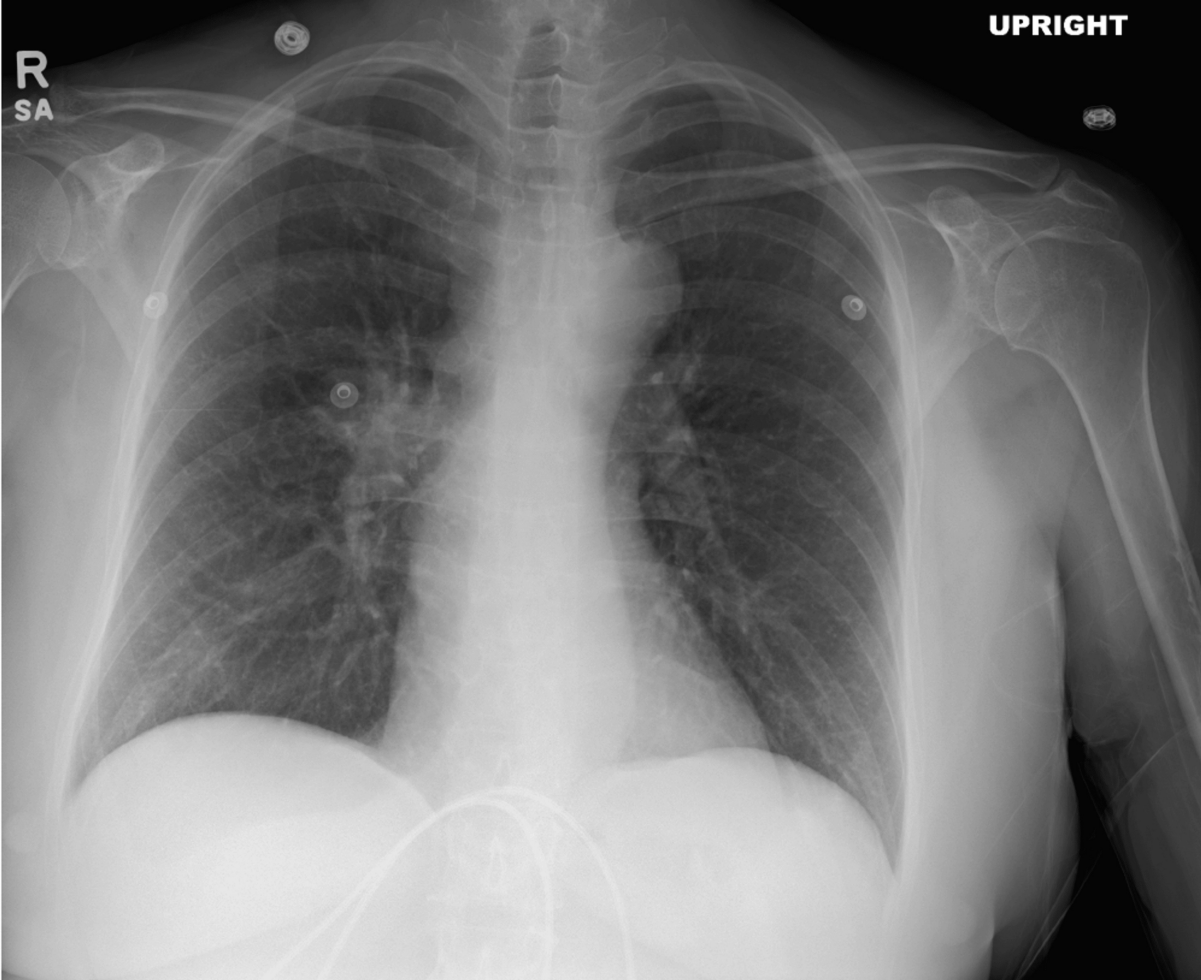
Chest X-ray from the third ED visit. Repeat chest X-ray demonstrating no evidence of acute cardiopulmonary disease, supporting no clinical change from prior airway findings.

After another week, the patient presented again with fever lasting several days, persistent fatigue, and continued concern over her previously elevated liver enzymes. She denied upper respiratory symptoms, urinary complaints, or gastrointestinal distress. On physical examination, she was alert and in no acute distress, with a temperature of 36.9°C, heart rate of 115 bpm, blood pressure of 133/75 mmHg, and oxygen saturation of 95%. Laboratory results showed persistent hyponatremia at 128 mmol/L (reference range: 135-146 mmol/L), hemoglobin of 10.6 g/dL (reference range: 12.0-16.0 g/dL), and alkaline phosphatase further elevated to 891 U/L (reference range: 44-147 U/L) (Table 1). Another chest X-ray again showed no findings (Figure 3). A CT abdomen/pelvis with contrast was performed, revealing small pleural effusions and mild atelectasis at the lung bases. No hepatic lesions, hepatomegaly, or biliary obstruction were identified to account for the elevated liver and cholestatic enzymes. Prior to presentation to the ED, she was seen by outpatient nephrology, where fluid restriction and sodium supplementation were recommended. She was advised to continue fluid restriction and sodium supplementation, with a follow-up recommendation to nephrology and discharged home with return precautions.

Subsequent visit #3 (ER visit #4, diagnosis of lung mass)

**Figure 4:**
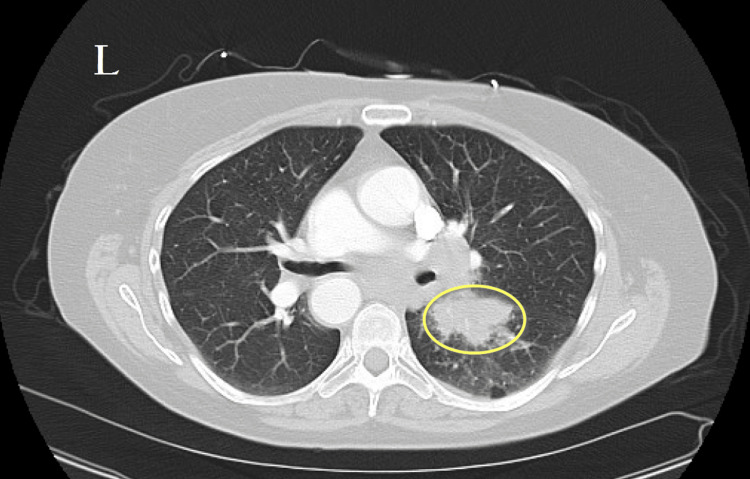
Contrast-enhanced CT of the chest. Contrast-enhanced CT of the chest demonstrating extensive right hilar lymphadenopathy and an adjacent irregular right perihilar pulmonary mass (yellow circle), highly suspicious for malignancy. Mediastinal lymphadenopathy is also present.

Three weeks later, the patient returned with worsening symptoms of fatigue, weakness, and near syncope, particularly after showers. She reported that her symptoms had been ongoing for over a month. On physical examination, she was alert but appeared fatigued. Her vital signs were notable for a blood pressure of 111/83 mmHg, heart rate of 90 bpm, respiratory rate of 16 breaths per minute, and oxygen saturation of 99%. Laboratory findings included a sodium level of 136 mmol/L (reference range: 135-146 mmol/L), hemoglobin of 10.6 g/dL (reference range: 12.0-16.0 g/dL), and D-dimer of 1,384 ng/mL (reference range: <500 ng/mL) (Table 1). A CT chest with IV contrast was performed for suspicion of a pulmonary embolism, revealing extensive right hilar lymphadenopathy with an adjacent irregular right perihilar pulmonary mass, highly suspicious for malignancy (Figure 4). Mediastinal lymphadenopathy was also noted. Given these findings, the patient was admitted for further workup.

Hospital course (hospital admission)

The patient was admitted for further evaluation of a suspected malignancy. Pulmonary consultation led to a scheduled endobronchial ultrasound-guided biopsy and navigational bronchoscopy. Immunocytochemistry was done, showing the tumor cells were positive for cytokeratin AE1/AE3, TTF-1, CD56, synaptophysin, and chromogranin and were negative for CK20 and Napsin-A, supporting the diagnosis of SCLC. On admission, she remained hemodynamically stable with persistent mild hyponatremia. Her hospital course was complicated by continued sodium abnormalities, managed with fluid restriction and sodium supplementation. Smoking cessation was strongly advised, and she was started on nicotine replacement therapy. The patient was ultimately discharged with plans for oncology follow-up and initiation of chemotherapy.

## Discussion

This case demonstrates the unique diagnostic challenge of SCLC presenting with a paraneoplastic syndrome before any radiographic evidence of malignancy was apparent. Despite multiple ED visits and a clear clinical pattern of persistent, unexplained hyponatremia, serial chest X-rays failed to reveal a pulmonary mass. Ultimately, the diagnosis was made only after a contrast-enhanced CT scan identified a suspicious hilar mass (Figure 4), reinforcing the importance of advanced imaging when clinical suspicion remains high.

Paraneoplastic syndromes occur in approximately 10-20% of all malignancies, and while paraneoplastic neurological syndromes are rare, SIADH is the most frequent paraneoplastic endocrine syndrome in SCLC, affecting up to 25% of these patients [9,10]. In fact, SCLC is the most common cause of cancer-related SIADH overall [9,10]. This case aligns with existing literature regarding the strong association between SIADH and SCLC, but it expands upon current understanding by illustrating how SIADH can precede radiographic evidence of malignancy. Most reports assume that the tumor is already apparent on imaging when SIADH develops [11-13], yet our patient’s experience suggests that secretory activity of SCLC can manifest before visible disease is detectable. This nuance underscores the importance of maintaining clinical vigilance in patients with unexplained or recurrent hyponatremia, particularly in those with a significant smoking history.

The diagnostic complexity in this case also highlights the multifactorial nature of hyponatremia in cancer patients. Drug-induced SIADH, volume depletion, and other paraneoplastic processes may coexist, making systematic exclusion of alternative causes essential. The diagnostic criteria for SIADH include euvolemic hypotonic hyponatremia, low serum osmolality, inappropriately high urine osmolality, elevated urine sodium, and exclusion of adrenal insufficiency, hypothyroidism, renal failure, and diuretic use [13,14]. Up to 60% of SIADH cases may be multifactorial or idiopathic, particularly in older adults [6,14,15], emphasizing the need for a careful and comprehensive diagnostic approach.

Management of SIADH in malignancy is typically guided by symptom severity. First-line therapy includes fluid restriction (<1 L/day), with vasopressin receptor antagonists (e.g., tolvaptan) or demeclocycline for refractory cases, and hypertonic saline in severe or symptomatic cases [13-17]. Importantly, definitive correction of hyponatremia often requires successful treatment of the underlying malignancy.

Finally, this case reinforces key clinical indicators that should prompt suspicion of an occult malignancy in patients with SIADH: new-onset or unexplained hyponatremia in older adults, significant smoking history, rapid symptom progression, or recurrence of hyponatremia after initial correction [6,18]. In SCLC, SIADH may present at diagnosis or recur with tumor relapse and is associated with a poorer prognosis.

In summary, this case aligns with existing literature by confirming the high prevalence of SIADH in SCLC and the diagnostic limitations of chest radiography in early disease. It expands upon current understanding by illustrating how SIADH can precede radiographic detection of malignancy, emphasizing the dynamic interplay between tumor biology and paraneoplastic syndromes. Recognizing these patterns and maintaining a high index of suspicion are crucial for early cancer detection and improved patient outcomes.

## Conclusions

This case explores the diagnostic challenges of SCLC when presenting with nonspecific, constitutional symptoms and persistent, unexplained hyponatremia. Despite multiple healthcare encounters and initial imaging with chest X-rays, the malignancy remained undetected until a chest CT revealed a right hilar mass. Clinicians should consider early use of advanced imaging in high-risk patients with unexplained hyponatremia, especially when paraneoplastic syndromes are suspected. This case reinforces the need for systematic evaluation and ongoing clinical vigilance in the diagnostic approach to SIADH.
